# Relationships between Mindfulness, Purpose in Life, Happiness, Anxiety, and Depression: Testing a Mediation Model in a Sample of Women

**DOI:** 10.3390/ijerph18030925

**Published:** 2021-01-21

**Authors:** Antonio Crego, José Ramón Yela, María Ángeles Gómez-Martínez, Pablo Riesco-Matías, Cristina Petisco-Rodríguez

**Affiliations:** 1 Department of Psychology, Pontifical University of Salamanca, Calle de la Compañía 5, 37002 Salamanca, Spain; jryelabe@upsa.es (J.R.Y.); magomezma@upsa.es (M.Á.G.-M.); priescoma@upsa.es (P.R.-M.); 2 Faculty of Education, Pontifical University of Salamanca, Calle Henry Collet 52-70, 37007 Salamanca, Spain; cpetiscoro@upsa.es

**Keywords:** mindfulness, purpose in life, behavioral activation, happiness, anxiety, depression

## Abstract

Mindfulness is connected to positive outcomes related to mental health and well-being. However, the psychological mechanisms that account for these relationships are largely unknown. A multiple-step multiple mediator structural equation modeling (SEM) model was tested with mindfulness as the independent variable; purpose in life and behavioral activation as serial mediators; and happiness, anxiety, and depression as outcome measures. Data were obtained from 1267 women. Higher mindfulness was associated with higher levels of happiness and lower anxiety and depression symptoms. The association of mindfulness with the outcome variables could be partially accounted for by purpose in life and behavioral activation. The SEM model explained large proportions of variance in happiness (50%), anxiety (34%), and depression (44%) symptoms. Mindfulness is associated with both a sense of purpose in life and engagement in activities, which are also connected with positive outcomes. Moreover, having purposes in life is linked to higher levels of behavioral activation.

## 1. Introduction

The positive effects of mindfulness have been highlighted by a growing number of publications. Mindfulness-based interventions have demonstrated efficacy in the treatment of depression, anxiety, and stress-related disorders, among many other psychological problems [1,2,3]. In addition, research has found that those people reporting higher levels of dispositional mindfulness also tend to experience positive states of mind and lower levels of depression and anxiety [4,5].

Essentially, mindfulness involves the self-regulation of attention so that it remains focused on the present moment. Along with this self-regulation, mindfulness also requires a focus on the immediate experience with an attitude of curiosity, openness, and acceptance [6]. How can this attention-related capacity lead to beneficial consequences in terms of mental health and well-being? Several mechanisms have been proposed. For example, it has been suggested that mindfulness practices could elicit processes of decentering, value clarification, exposure, cognitive/behavioral flexibility, and self-management [7,8,9,10]. Others propose attention monitoring and acceptance of inner experiences [11]; emotional intelligence [5]; self-compassion [7,12]; and self-regulation of processes such as attention, increased body awareness, emotional regulation through reappraisal, exposure, extinction and reconsolidation, and change in one’s perspective of the self [13,14]. However, empirical testing of such explanatory mechanisms is still scarce, and results are often not conclusive. In this research, we argue that two variables, purpose in life and behavioral activation, may be involved in the association between mindfulness and salutary outcomes. Thus, we aimed at testing an empirically supported model intended to shed light on how mindfulness is connected with beneficial effects on mental health and happiness. In particular, we propose a link between mindfulness and having purposes in life, a variable expected to be associated with greater behavioral activation.

### 1.1. Purpose in Life and Behavioral Activation as Mediators between Mindfulness and Outcomes

Purpose in life may be defined as an elementary aim that serves to self-organize and inspire concrete goals, trigger behaviors, and provide meaningfulness assessments [15]. Having life purposes and valuable aims that orient one’s behavior and provide a sense of direction is mentioned as a key ingredient of a meaningful life [16]. Like mindfulness, the construct of meaning in life comprises several components. The presence of meaning in life entails being able to identify abstract, relevant, and long-term goals that guide more specific objectives and behaviors. Furthermore, a meaningful life also requires having a sense of understanding or comprehension of life, being committed to one’s personal values and that which is personally important, and a belief that life matters and is worth it, despite unavoidable suffering and pain [17,18].

Interestingly, the presence of meaning and purpose in life has been found to be positively associated with mindfulness. For instance, increased mindfulness has been found to be positively associated with greater presence of meaning in life, a relationship that could be explained by increased self-awareness [19], and greater knowledge of and trust in oneself and awareness of one’s strengths and weaknesses [20]. Similarly, the processes of cognitive reappraisal, wise evaluation, and discernment, which are entailed in mindfulness, have been suggested to explain assessments of life as meaningful and purposeful, the appreciation of important things, and posttraumatic growth in the face of adverse events of life [21,22].

Support for the connections between mindfulness and increased purpose in life comes not only from correlational research but also from experimental studies. For instance, Jacobs et al. [23] found that individuals who attended a meditation retreat on training attention skills and benevolent mental states (i.e., loving-kindness, compassion, and equanimity) showed significantly greater increases in purpose in life than controls. Similarly, Bloch et al. [24] reported increased levels of the presence of meaning in life following a meditation course comprising mindfulness practices and cultivation of loving kindness and compassionate attitudes towards oneself and others in a sample of undergraduate college students.

Purpose in life has been proposed to be a central construct to explain well-being- and health-related outcomes [15]. People engaged in a purposeful and meaningful life tend to report positive outcomes in terms of well-being and mental health. The presence of meaning in life appears to be linked to happiness, life satisfaction, and positive affect [25,26,27,28,29,30,31,32,33]. Moreover, having purposes in life has been found to be negatively connected with various psychopathological problems [34,35], such as depressive symptoms [9,36], suicidal ideation [37,38], anxiety-related responses [39], post-traumatic stress disorder [40], social anxiety [41], and sleep disturbances [42], among other issues. Higher levels of purpose in life could be even connected to better physical health outcomes, through enhanced functioning of physiological systems [23,35]. Interestingly, previous research has found that meaning in life mediated the relationships between mindfulness and positive and negative wellbeing [43].

Having purposes in life is somehow considered a motivation to greater behavioral activation. The construct of meaning in life entails a motivational component [17,18], which drives people to attain valuable aims and persevere in significant behavior in spite of potential obstacles [15]. From a theoretical perspective, purposeful living has been suggested to increase endurance during mentally and physically challenging activities and sustain vitality [15]. In addition, empirical research has reported strong positive associations between behavioral activation and three components of meaning in life, i.e., comprehension, purpose, and mattering [44]. Concerning mental health outcomes, interventions focused on increasing behavioral activation have demonstrated large positive effects on depression symptoms [45,46,47], shown moderate effects on well-being [48], and have been suggested as an evidence-based mechanism for anxiety exposure [49].

Behavioral activation may somewhat explain the relationship between purpose in life and potential mental health and well-being-related outcomes. First, life purposes operate as a motivational component [18]. Individuals striving to approach and achieve life aims seem to engage in significant behavior [15]. Second, positive correlations between mindfulness and behavioral activation have been reported [50,51,52]. Moreover, Kearney et al. [53,54] found that posttraumatic stress disorder (PTSD) patients who completed a mindfulness-based stress reduction (MBSR) intervention increased their levels of behavioral activation. Interestingly, they showed that changes in mindfulness scores were associated with change in behavioral activation [53]. Similarly, Gaudiano et al. [55] reported a strong correlation between changes in mindfulness and changes in behavioral activation, following an acceptance-based depression and psychosis therapy (ADAPT) intervention in patients with major depressive disorder with psychotic features.

### 1.2. The Present Study

On the basis of previous literature on mindfulness, purpose in life, behavioral activation, and well-being and mental health-related outcomes, our study aimed to test a model that integrates the complete patterns of relationships among these variables. This model entails the following hypotheses:

**Hypothesis 1 (H1).**  *Mindfulness is expected to be positively correlated with happiness (H1.1) and negatively associated with anxiety (H1.2) and depression (H1.3) measures.*

**Hypothesis 2 (H2).**  *Higher levels of mindfulness will be connected with a greater sense of purpose in life (H2.1) and behavioral activation (H2.2).*

**Hypothesis 3 (H3).**  *Purpose in life will be positively connected with happiness (H3.1) and negatively associated with anxiety (H3.2) and depression (H3.3) measures.*

**Hypothesis 4 (H4).**  *Higher behavioral activation is expected to be associated with higher levels of happiness (H4.1) and lower scores in anxiety (H4.2) and depression (H4.3).*

**Hypothesis 5 (H5).**  *Higher levels of purpose in life are expected to correspond to higher levels of behavioral activation (H5).*

Taken as a whole, the abovementioned hypotheses suggest some possible mediating effects that will be tested:

**Hypothesis 6 (H6).**  *The associations between mindfulness and happiness (H6.1), anxiety (H6.2), and depression (H6.3) may be accounted for, to some extent, by both purpose in life and behavioral activation.*

**Hypothesis 7 (H7).**  *The link between mindfulness and behavioral activation may be accounted for, to some extent, by purpose in life (H7).*

**Hypothesis 8 (H8).**  *The associations between purpose in life and happiness (H8.1), anxiety (H8.2), and depression (H8.3) may be accounted for, to some extent, by behavioral activation.*

## 2. Materials and Methods

### 2.1. Participants

Our sample comprised 1267 women. Their mean age was 33.76 years (*SD* = 14.85) and ranged from 18 to 70 years. Eighteen Latin American Spanish-speaking countries and Spain were represented. Most of the participants came from Venezuela (44.2%), Nicaragua (10.3%), Bolivia (8.4%), Paraguay (5.6%), the Dominican Republic (5.4%), and Argentina (5.2%). Other nationalities represented less than 5%. Concerning education, 14% of the participants reached the postgraduate level, and 52.2% completed graduate studies. Professional training and high-school education represented, respectively, 15.4% and 16.3%, with elementary studies being reported by 1.9% of the sample. More than one-third of the participants (38.8%) were active workers, 14.1% were unemployed, 32% were students without a job allowing for economic autonomy, 7.7% were retirees, and 7.3% reported other labor situations. With respect to the participants’ attitudes toward religion, most (56.2%) characterized themselves as “nonpracticing believers”; 27.7% reported being believers involved in religious practice; and 16.1% defined themselves as atheists, agnostics, or indifferent concerning religious belief.

### 2.2. Procedure

An online questionnaire was used to collect data from May to July 2018. The respondents were informed that this study was part of a research project aiming to know, from a psychological perspective, more about meaning in life, mindful living, health, and well-being. The questionnaire did not request any data allowing for individual identification of participants. Before starting the survey, the respondents were also informed that all data would be anonymous. A snowball method was used to distribute questionnaires through online social networks, encouraging participants to share the link to the survey webpage among their acquaintances.

Completing the questionnaire was voluntary, with no monetary or material compensation or other incentive for participants. Informed consent was obtained for all participants. All procedures performed in this study were done in accordance with the ethical standards as laid down in the 1964 Declaration of Helsinki and its later amendments. This research received approval from the Research Ethics Committee of the Pontifical University of Salamanca (Minutes of the meeting 17 July 2018). In total, 1528 questionnaires were received. However, 155 of them were discarded because they were duplicated submissions (57 questionnaires) or responses out of the age range of this research, i.e., younger than 18 years (82 questionnaires) or older than 70 years (16 questionnaires). Questionnaires from male individuals (106 submissions) were not considered in this study in order to avoid potential problems due to the unequal distribution of gender among respondents. Therefore, female respondents participating in this study represent 82.92% of the received questionnaires.

### 2.3. Instruments

Mindfulness. The participants’ capacity for paying attention to experiences and being fully aware of internal and external stimuli while being focused in the present moment was measured by means of the Mindful Attention Awareness Scale (MAAS), developed by Brown and Ryan [56] (Spanish adaptation by Soler et al. [57]). This scale comprises 15 items designed to measure the individual’s general mindfulness capacity. It uses a 6-point Likert-type response format where 1 = “Almost always” and 6 = “Almost never”. An example item is “I find it difficult to stay focused on what’s happening in the present”. Total scores were obtained by averaging each participant’s responses to the items, with higher scores meaning a greater mindfulness capacity. Internal consistency was α = 0.88.

Purpose in life. The Spanish version, developed by Díaz et al. [58], of the Purpose in Life Scale included in the Ryff’s Psychological Well-being Scales was used [59]. This 5-item scale aims to assess how much the respondents feel their lives are purposeful and meaningful. An example item is “I have a sense of direction and purpose in life”. Participants responded on a 6-point Likert-type scale, where 1 = “Totally disagree” and 6 = “Totally agree”. A total score for each participant was calculated by averaging the 5 items of the subscale, with higher scores representing higher levels of purpose in life. The internal consistency reliability for the purpose in life items was α = 0.87.

Behavioral activation. The 7-item “Activation” subscale of the Behavioral Activation for Depression Scale (BADS), developed by Kanter et al. [60] (Spanish adaptation by Barraca et al. [61]) was used. These items measure focused, goal-directed activation and completion of planned activities, e.g., “I did things even though they were hard because they fit in with my long-term goals for myself”. Responses are made on a 7-point Likert-type scale from 0 = “Not at all” to 6 = “Completely”. Items were averaged to obtain a total score for each participant. Higher scores reflect a higher level of behavioral activation. Internal consistency was α = 0.92.

The Subjective Happiness Scale (SHS) is a 4-item scale used to measure the global level of perceived happiness [62]; Spanish translation of items and wording of response labels have been done by Extremera and Fernández-Berrocal [63]. Two items ask respondents to report the extent to which they consider themselves to be a happy or not-happy person, in absolute terms and relative to other people (e.g., “Compared to most of my peers, I consider myself: less happy/more happy”). The other two items present descriptions of happy and unhappy people, and respondents are requested to indicate the extent to which each description applies to themselves (e.g., “Some people are generally very happy. They enjoy life regardless of what is going on, getting the most out of everything. To what extent does this characterization describe you?: not at all/a great deal”). All items use a 7-point Likert-type scale. The total scores of subjective happiness were calculated for each participant by averaging responses to the 4 items (range 1–7), with higher scores indicating greater subjective happiness. The internal consistency reliability was α = 0.85.

Anxiety and depression symptoms. The Hospital Anxiety and Depression Scale (HAD), developed by Zigmond and Snaith, was used [64] (Spanish adaptation by Terol et al. [65]). This scale comprises 14 items intended as a screening instrument to detect possible anxiety (7 items) and depression (7 items problems). Examples items are “I get sudden feelings of panic” (anxiety) and “I have lost interest in my appearance” (depression). Participants respond by selecting 1 of 4 alternatives that are scored from 0 to 3. Scores for anxiety and depression are calculated by adding the individual’s responses to the items in each subscale, with higher scores indicating higher levels of anxiety and depression. The internal consistency values were α = 0.85 (anxiety subscale) and α = 0.76 (depression subscale).

### 2.4. Data Analyses

Descriptive statistics (means and standard deviations) were calculated for the study variables. Pearson’s *r* bivariate correlations were obtained to assess the associations among variables.

Following Hayes [66,67], bootstrap confidence intervals are preferred to normal theory tests for inference about indirect effects (i.e., the mediation effects). Therefore, a bootstrapping-based method was used to test mediation effects. Point estimates and 95% bias-corrected (*BC*) bootstrap confidence intervals (percentile method) for the indirect (mediated) effects were calculated using 5000 bootstrap samples. A statistically significant mediated effect, different from zero with 95% confidence, is obtained if zero is not between the lower and upper bound of the *BC* confidence interval for the indirect effects. The ratio of the indirect effect to the total effect (*P_M_*) was calculated as an effect-size measure for mediation effects. Although this ratio cannot be properly interpreted in terms of the proportion of the explained variance in total effects that is due to the indirect effect [68], it is usually reported as an index of the relative magnitude of the mediation path.

**Figure 1:**
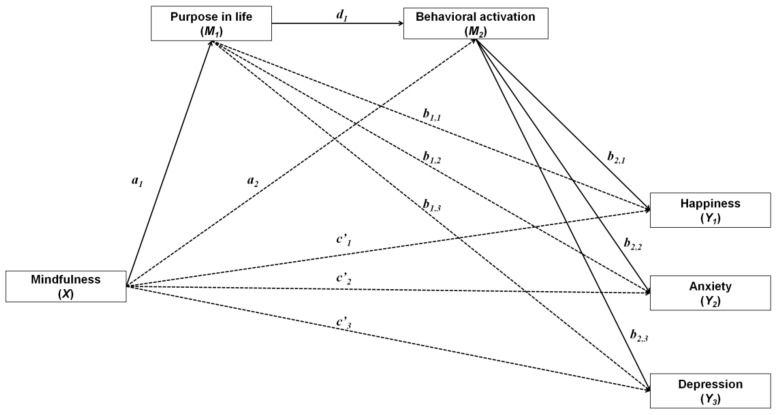
Hypothesized multiple-step multiple mediator model.

Our hypotheses entailed 2 mediators, purpose in life (*M*_1_) and behavioral activation (*M*_2_), and 3 outcome variables: happiness (*Y*_1_), anxiety (*Y*_2_), and depression (*Y*_3_). Therefore, a multiple-step multiple mediator model was used [66,67] (Figure 1). A structural equation modeling (SEM)-based approach was used to test the hypothesized effects. SEM modeling allows for testing mediation effects simultaneously, with the advantage of identifying whether a particular mediation is independent of the effect of the other mediators [69]. Because the mediation models are saturated (i.e., zero degrees of freedom), the Akaike Information Criteria (AIC) was used to compare the adequacy of the proposed model against alternative models where *a*, *b*, and/or *c’* paths were omitted [70].

Prior to conducting the analyses, quantitative variables were standardized to avoid possible multicollinearity problems because correlations among our study variables were expected. With the values of the variance inflation factor (VIF) below 10, tolerance values higher than 0.2, and condition indexes clearly below 15, allowed us to safely conclude that there was no collinearity in the data, according to the usual criteria [71,72].

All analyses were carried out using the statistical package IBM SPSS 19 and AMOS 16 (IBM, Armonk, NY, USA).

## 3. Results

Participants presented moderate levels of mindfulness, with average scores around the midpoint of the response scale (Table 1). Similarly, they reported a moderate presence of purpose in life and behavioral activation. Concerning anxiety scores, overall, participants obtained an average score of 9.19 (*SD* = 4.36), which yielded a 95% CI with 8.95 and 9.43 as lower and upper limits, respectively. According to Bjelland et al. [73], a cut-off point ≥8 may be indicative of anxiety and depression symptoms as measured by the corresponding HAD subscales. Therefore, on average, respondents presented moderate or borderline anxiety-related symptoms. Depression scores were rather low, and clearly below the 8 point cut-off. Finally, given the possible range of scores on the scale, an intermediate average level of subjective happiness was observed among participants.

Age was moderately correlated with all of the focus variables of interest, yielding positive associations with mindfulness, purpose in life, behavior activation, and happiness, as well as presenting negative relationships with anxiety and depression (Table 2).

As presented in Table 2, the results revealed strong bivariate correlations among the main study variables. Mindfulness was strongly and positively connected to higher happiness (H1.1) and negatively associated with anxiety (H1.2) and depression (H1.3), with percentages of shared variances of 22.09%, 23.04%, and 19.36%, respectively. Higher levels of mindfulness corresponded to greater perceptions of purpose in life (H2.1) and behavioral activation (H2.2) with 21.16% and 15.21% of shared variance between these variables and mindfulness, respectively. In addition, experiencing a purposeful life was linked to positive outcomes, such as increased happiness (H3.1) and reduced anxiety (H3.2) and depression (H3.3) symptoms, with percentages of shared variance ranging from 46.24% to 25%. Behavioral activation was also strongly associated with the outcome variables. The higher the behavioral activation reported, the higher the reported happiness (H4.1) scores and the lower the symptoms-related scores (H4.2 and H4.3). The percentage of shared variance between happiness and behavioral activation was 31.36%, and depression and anxiety yielded percentages of 32.49% and 20.25%, respectively, of shared variations with behavioral activation. Participants reporting a highly meaningful and purposeful life also reported higher levels of completion of planned and goal-directed behavior (H5), with 50.41% of shared variance between purpose in life and behavioral activation.

Finally, as expected, happiness was negatively and statistically significantly associated with anxiety and depression, whereas significantly positive correlations between psychological symptoms were observed.

### 3.1. Purpose in Life and Behavioral Activation as Mediators of the Associations between Mindfulness and Outcome Variables

After controlling for the effects of age, mindfulness scores significantly predicted happiness, anxiety, and depression, thus confirming the existence of significant total effects that may be mediated (Table 3). Mindfulness was also a significant predictor of the two proposed mediators, with significant total effects on purpose in life and behavioral activation, after controlling for age (Table 3).

The proposed mediators also yielded significant total effects on the outcome variables (Table 3). After controlling for age and mindfulness, purpose in life was a significant predictor of happiness, anxiety, and depression. Similarly, behavioral activation predicted happiness, anxiety, and depression, after controlling for age, mindfulness, and purpose in life. 

Bootstrap-corrected confidence intervals for the indirect effects of mindfulness through purpose in life and behavioral activation confirmed significant mediation effects on happiness (H6.1), anxiety (H6.2), and depression (H6.3) (Table 4).

### 3.2. Purpose in Life as a Mediator of the Relationship between Mindfulness and Behavioral Activation

The abovementioned significant connection between mindfulness and behavioral activation was proposed to be mediated through purpose in life (H7). Here, two additional conditions were met. As presented above, mindfulness was also associated with purpose in life. Second, purpose in life yielded a significant total effect on behavioral activation (Table 3), after controlling for age and mindfulness. The bootstrap bias-corrected 95% confidence interval for the indirect effect confirmed a significant mediation effect of purpose in life on the relationship between mindfulness and behavioral activation (Table 4).

### 3.3. Behavioral Activation as a Mediator of the Associations between Purpose in Life and Outcome Variables

As previously mentioned, purpose in life was a significant predictor of happiness, anxiety, and depression after controlling for age and mindfulness. This relationship was hypothesized to be mediated by behavioral activation. The required conditions were met because purpose in life was a significant predictor of behavioral activation after controlling for age and mindfulness; additionally, behavioral activation significantly predicted the outcome variables (i.e., happiness, anxiety, and depression), after controlling for age, mindfulness, and purpose in life. Bootstrap bias-corrected 95% confidence intervals indicated a significant indirect (mediated) effect of purpose in life on the outcome variables through behavioral activation (Table 4), thus supporting H8.1, H8.2, and H8.3.

**Figure 2:**
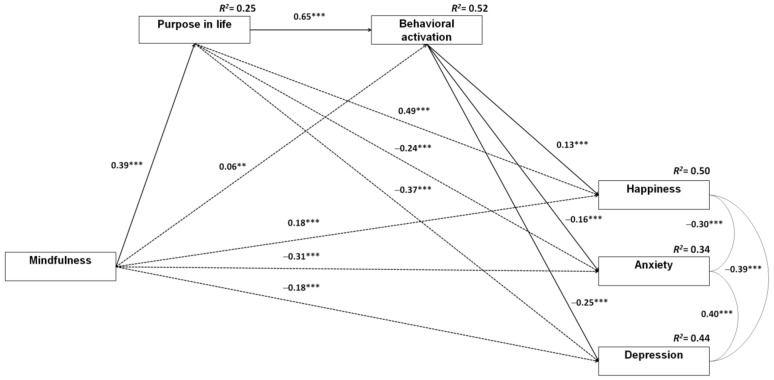
Multiple-step multiple mediator model representing the associations between mindfulness and happiness, anxiety, and depression, with purpose in life and behavior as mediators. Notes: The numbers represent point estimates of direct effects. Dashed lines represent mediated paths. ** *p* < 0.01; *** *p* < 0.001. Control variable (i.e., age) is not depicted to provide a clear representation of the model.

The saturated SEM model of the multiple-step multiple mediation (Figure 2) always fit better (AIC = 70.00) than alternative models where paths *a*, *b*, or *c’* were omitted.

With respect to the amount of variance in the outcomes variables that were accounted for by the multiple-step multiple mediator model, 50%, 34%, and 44% of variations in happiness, anxiety, and depression, respectively, were explained.

## 4. Discussion

Our study contributes to shedding light on the potential mechanisms that may connect mindfulness and healthy outcomes. We found that individuals who reported higher levels of mindfulness also showed higher levels of happiness and lower anxiety and depression symptoms. In this regard, the negative correlations found between mindfulness and depression and anxiety are highly consistent with correlations previously found [4,56,74]. Associations between mindfulness and happiness have also been previously reported [5,74].

How is mindfulness connected with positive outcomes? We hypothesized a series of sequential associations between mindfulness, having purposes in life, and increased behavioral activation. In this regard, mindfulness may lead people to clarify values around which to organize their lives and gain awareness of those things that truly matter in life, i.e., to discern their purposes and find meaning in life. Consistent with previous research, we found that individuals reporting higher levels of mindfulness also reported a greater sense of purpose in life [9,43,75]. However, why might mindfulness stimulate a person to set life aims? Some new hypotheses may be suggested. First, enhanced awareness of difficult thoughts and painful emotions (i.e., mindfulness), paralleled by a benevolent and nonjudgmental attitude towards oneself, may favor wise discernment, identification of personal values, and disclosure of paths to behavioral activation and persistence in the face of adverse events. As mentioned, previous research has already linked mindfulness skills and life purpose assessments (e.g., Allan et al. [19]). Second, mindfulness-related processes and attitudes, such as decentering, acceptance, openness, and not judging, could reduce the extent to which individuals are exposed to self-punishment, blame, shame, and other self-directed negative emotions, which could lead to greater appreciation of life and savoring of things that matter, i.e., increasing meaningfulness.

Purpose in life and the outcome variables were connected as expected. The association between having purposes and happiness was coincident with Lyubomirsky et al. [76] and García-Alandete [26], as well as being consistent with correlations between meaning in life and happiness reported by Steger et al. [33] and Schueller and Seligman [77]. The association found between purpose and depression was also similar to that reported in previous research [33,78,79]. Concerning anxiety, a similar correlation to ours was reported by Ishida and Okada [80]. Our results concerning the relationships between purpose in life and depression and anxiety are also consistent with those of Ho et al. [81], Schnell [31], Scheier et al. [82], and Pearson et al. [9], although lower correlations were obtained by these authors.

Interestingly, Pearson et al. [9] tested a path model where purpose in life was proposed to mediate the relationships among trait mindfulness and anxiety and depression symptoms. They found that purpose in life actually mediated the association between mindfulness and depression. However, purpose in life had no significant direct effect on anxiety symptoms, as another mediator, i.e., decentering, appeared to better account for the association between mindfulness and anxiety. These results may indicate that, while purpose in life is highly relevant to explain mood-related and well-being related outcomes, it may play a different role concerning anxiety symptoms. In our research, we found significant associations (as measured by correlations and total effects) between purpose in life and anxiety scores. However, in terms of effect size and percentage of explained variance, our model also revealed that the associations of purpose in life with happiness and depression were stronger than the connection between having purposes and anxiety.

This research also contributes to extend previous models where meaningfulness is considered to mediate the positive effects of mindfulness [43] by adding a new variable, i.e., behavioral activation. We hypothesized that mindfulness will be connected with the individual’s activation, a relationship that could be partially explained by the links between purposefulness with both mindfulness and behavioral activation. In this regard, the positive correlation found between mindfulness and behavioral activation was coherent with previous findings [50,51,52]. Furthermore, our results indicate that such connection between mindfulness and activation may be explained, to a great extent, by higher levels of purposefulness. The more a person perceives his or her life has a purpose and envisions valuable aims to pursue, the more prone to engage with activities he or she will be. Previous research has already found a positive connection between purpose in life and behavioral activation [44], which is consistent with our results. As presented, purpose in life is assumed to play a motivational role that prompt exposure to experience [7,15,18]. Finally, our results concerning the association of behavioral activation with anxiety, depression, and happiness are also aligned with previous studies [47,48,52,83].

In summary, higher levels of mindfulness and purpose in life appears to be connected with greater engagement with life, which is linked to beneficial psychological outcomes.

### 4.1. Limitations and Future Research

This research’s contributions should be considered in the light of its limitations. First, although relationships among variables depicted in SEM models apparently suggest causality, especially when mediation analyses are tested, such causal inferences cannot be properly derived from cross-sectional data, as used in this research. Therefore, assertions that may suggest a possible direction of causality should be taken cautiously. In this regard, longitudinal designs and cross-lagged analyses would be advisable in future research. Research exploring the effects of mindfulness-based interventions would offer interesting possibilities to longitudinally analyze whether changes in mindfulness-related attitudes may lead to changes in meaningfulness and changes in engagement with life, which would be expected to be associated with changes in wellbeing and mental health across time. A second limitation comes from the sample participating in this study. As stated, we used a convenience sample, recruited among internet users (i.e., the survey was distributed online), which may compromise the generalization of the results. In particular, our study used a sample of women. Previous research has found that women usually report higher levels of anxiety [84] and depression [85]. Although a comparatively small number of men responded to the online survey, their data were not used in the analyses to avoid potential gender biases in the analyses performed, due to the imbalance that would have occurred in the overall sample. While this is an obvious limitation, several previous studies have also pointed out the unequal participation rates for males and females in online surveys [86,87]. Despite this limitation, our results concerning the relationships among variables were highly consistent with findings from previous research. In any case, a possible line of future research would be analyzing the influence of gender on the relationships among mindfulness, purpose in life, behavioral activation, mental health and happiness, and testing of whether the proposed model is also applicable to the male population. Previous literature has also suggested the convenience of paying attention to gender in the context of mindfulness research [88]. Likewise, an in-depth analysis of the validity of the model in different age groups would also be interesting.

Finally, we considered two mediating mechanisms that, along with mindfulness, could account for the great proportions of the variance of happiness, anxiety, and depression. However, our mediating variables do not exhaust other potential explanatory mechanisms that may be involved in the relationship between mindfulness and psychological outcomes. Similarly, additional mediating and/or moderating variables could be explored concerning the relationships among mindfulness and purpose in life and the connections between purpose and behavioral activation. In this regard, research focused on integrating new possible explanatory variables into the proposed model would be worth doing. For instance, it would be interesting to analyze the role played by personality traits, and eventually include this variable in the model, since previous research has found that part of the variance in meaningfulness can be explained by personality factors [89]. In addition, it would be also interesting to know how contextual factors may affect the relationships between variables. Our data collection was conducted prior to the emergence of the global coronavirus disease 2019 (COVID-19) pandemic, which has been a major stressor for many people. A global event of this magnitude may have prompted many individuals to reflect on their life purposes and identify values that make life meaningful. On the other hand, the lockdowns occurring in many countries may have represented a challenge to maintaining adequate levels of behavioral activation. For these reasons, it would be interesting to test how robust the proposed model is in exceptional situations, such as those experienced as a result of the COVID-19 pandemic.

### 4.2. Practical Implications

Our results may contribute to provide some rationale for intervention approaches that include the training of mindfulness skills. As presented, mindfulness is associated with positive psychological outcomes. Moreover, this study contributes to shed light on how such association may be accounted for. Having a sense of purpose in life and behavioral activation appeared to play a relevant role on the connections between mindfulness and happiness, anxiety, and depression. This point yields important possibilities for clinical practice. For instance, clinicians using mindfulness-based approaches could presumably strengthen the links between mindfulness and positive outcomes by focusing on promoting a sense of purpose and fostering involvement in meaningful activities, as such variables are associated to lower anxiety and depression symptoms and higher happiness. In fact, evidence-based approaches such as acceptance and commitment therapy [90] and the mindful self-compassion protocol [91] already include, in addition to working on mindfulness skills, sessions aimed at promoting meaningful purposes and engagement in actions that are valuable for the individual.

## 5. Conclusions

Mindfulness, meaningfulness, and behavioral activation are connected with enhanced happiness and reduced anxiety and depression symptoms. Moreover, mindfulness is linked to increased sense of purpose in life, which was revealed to be associated with greater activation and engagement with valued activities. To some extent, the relationships between mindfulness and salutary outcomes could be accounted for by factors such as meaningfulness and behavioral activation, which were identified as mediating variables. Here, the findings may contribute to suggest psychological pathways aiming to reduce mental health disturbances and promote a happy life.

## Figures and Tables

**Table 1:** Descriptive statistics for mindfulness, the proposed mediators (purpose in life and behavior activation), and outcome variables (anxiety, depression, and happiness).

	Range of Scores	Mean	*SD*	95% Confidence Interval of the Mean	
				Lower	Upper
Mindfulness	1–6	3.87	1.06	3.81	3.92
Purpose in life	1–6	4.07	1.33	4.00	4.14
Behavioral activation	0–6	3.57	1.63	3.48	3.66
Anxiety	0–21	9.19	4.36	8.95	9.43
Depression	0–21	6.14	3.98	5.92	6.36
Happiness	1–7	4.44	1.48	4.36	4.52

**Table 2:** Bivariate correlations among the study variables.

	1	2	3	4	5	6
1. Age						
2. Mindfulness	0.31					
3. Purpose in life	0.33	0.46				
4. Behavioral activation	0.34	0.39	0.71			
5. Happiness	0.31	0.47	0.68	0.56		
6. Anxiety	−0.22	−0.48	−0.50	−0.45	−0.57	
7. Depression	−0.25	−0.44	−0.62	−0.57	−0.67	0.62

Note: All correlations were significant at the *p* < 0.001 level (two-tailed).

**Table 3:** Standardized total effects of mindfulness, purpose in life, and behavior activation on mediators and outcome variables.

	Paths	Point Estimate	95% BC CI
Total effects of mindfulness on			
Happiness	*c’*_1_*+ a*_1_ × *b*_1,1_ *+ a*_2_ × *b*_2,1_ *+ a*_1_ × *d*_1_ × *b*_2,1_	0.41	[0.36, 0.46]
Anxiety	*c’*_2_*+ a*_1_ × *b*_1,2_ *+ a*_2_ × *b*_2,2_ *+ a*_1_ × *d*_1_ × *b*_2,2_	−0.46	[−0.50, −0.41]
Depression	*c’*_3_*+ a*_1_ × *b*_1,3_ *+ a*_2_ × *b*_2,3_ *+ a*_1_ × *d*_1_ × *b*_2,3_	−0.40	[−0.45, −0.35]
Purpose in life	*a*_2_	0.39	[0.34, 0.44]
Behavioral activation	*a*_2_*+ a*_1_ × *d*_1_	0.31	[0.25, 0.37]
Total effects of purpose in life on			
Happiness	*b*_1,1_*+ d*_1_ × *b*_2,1_	0.57	[0.52, 0.61]
Anxiety	*b*_1,2_*+ d*_1_ × *b*_2,2_	−0.35	[−0.40, −0.29]
Depression	*b*_1,3_*+ d*_1_ × *b*_2,3_	−0.53	[−0.57, −0.48]
Behavioral activation	*d*_1_	0.65	[0.60, 0.69]
Total effects of behavioral activation on			
Happiness	*b*_2,1_	0.13	[0.07, 0.20]
Anxiety	*b*_2,2_	−0.16	[−0.24, −0.09]
Depression	*b*_2,3_	−0.25	[−0.31, −0.18]

**Table 4:** Standardized indirect effects of mindfulness and purpose in life on mediators and outcome variables.

	Paths	Point Estimate	95% BC CI	Ratio IE/TE
Mindfulness through purpose in life on				
Behavioral activation	*a*_1_ × *d*_1_	0.25	[0.22, 0.29]	0.81
Mindfulness through purpose in life and behavioral activation on				
Happiness	*a_1_* × *b*_1,1_ *+ a*_2_ × *b*_2,1_ *+ a*_1_ × *d*_1_ × *b*_2,1_	0.23	[0.20, 0.26]	0.56
Anxiety	*a*_1_ × *b*_1,2_ *+ a*_2_ × *b*_2,2_ *+ a*_1_ × *d*_1_ × *b*_2,2_	−0.14	[−0.18, −0.12]	0.30
Depression	*a*_1_ × *b*_1,3_ *+ a*_2_ × *b*_2,3_ *+ a*_1_ × *d*_1_ × *b*_2,3_	−0.22	[−0.26, −0.19]	0.55
Purpose in life through behavioral activation on				
Happiness	*d*_1_ × *b*_2,1_	0.08	[0.04, 0.13]	0.14
Anxiety	*d*_1_ × *b*_2,2_	−0.10	[−0.16, −0.06]	0.29
Depression	*d*_1_ × *b*_2,3_	−0.16	[−0.20, −0.12]	0.30
